# The virtual COVID-19 classroom: surveying outcomes, individual differences, and technology use in college students

**DOI:** 10.1186/s40561-021-00174-7

**Published:** 2021-11-01

**Authors:** Kara Sage, Sophia Jackson, Emily Fox, Larissa Mauer

**Affiliations:** The College of Idaho, 2112 Cleveland Blvd, Caldwell, ID 83605 USA

**Keywords:** Online classes, Digital media, Smartphones, Postsecondary education, Learning

## Abstract

The COVID-19 pandemic caused many colleges to quickly shift to virtual learning, leading students to rely on technology to complete coursework while also experiencing new situations and stressors. The present study explored students’ technology use in their online course in conjunction with several student outcomes and individual difference measures. Ninety-six undergraduate students were surveyed about devices used and their perceptions of those devices. In addition, the survey measured students’ engagement, motivation, procrastination, perceived stress, and self-efficacy. It also asked students to report their current grade as well as how satisfied and isolated they felt in their course. Relationships emerged in predictable ways between course outcomes and individual difference measures. And though laptops were most used for coursework, more smartphone use related to lower feelings of isolation. Lower feelings of isolation then related to higher grades and less stress. Regression analyses confirmed that smartphone use explained unique variance in feelings of isolation, and further revealed that perceived stress consistently predicted all outcomes. From these results and complementary qualitative survey data, it seems that both laptops and smartphones hold importance for academics in the current context. Educators should further explore the role of device in students’ experience as well as consider this information when designing online courses.

## Introduction

The onset of the COVID-19 (coronavirus disease 2019) pandemic in the United States caused abrupt changes to the landscape of higher education. Faced with an indefinite timeframe and health measures such as quarantines, a shift to virtual learning was deemed a necessary measure to balance safety with education needs. Though the shift occurred quickly, ownership of mobile devices was already ubiquitous among the college student population (e.g., Gierdowski, 2019; Sage et al., 2020). Most students today own both smartphones and laptops, with smartphones often being the most popular as students find them convenient and helpful for communication (e.g., Anshari et al., 2017; Sage et al., 2020). Though students already use both technologies for educational purposes (e.g., Callaghan, 2018; Stec et al., 2018), the pandemic created an immediate need to use those digital technologies specifically to attend classes and meet with professors. These technologies vary in their affordances. They differ perceptually (e.g., text size), cognitively (e.g., ability to focus), and physically (e.g., weight or interface). Thus, it stands to reason that different technologies may affect students in distinct ways when used for online coursework.

The present research explored students’ technology choices, experiences, and perceptions while taking an online course necessitated by the COVID-19 pandemic. Their experiences were then related to and discussed in the context of various student outcomes and individual difference measures, such as feelings of isolation, engagement, and perceived stress. Though research has repeatedly explored online course experiences (e.g., Ralston-Berg et al., 2015), little research has focused on the physical technology students use to interact with their courses, and not during a global pandemic. Such research can help predict the challenges encountered by teachers and students adapting to the online educational environment during the COVID-19 pandemic as well as provide recommendations for these online spaces.

### Online course quality and frameworks

Online courses have existed for a few decades and have been extensively examined in terms of their quality. One frequently cited set of research-based standards for online courses is *Quality Matters* (https://www.qualitymatters.org/). By reviewing these standards, educators at higher education institutions can determine if their course design meets a particular quality threshold (Ralston-Berg et al., 2015). The *Quality Matters* (QM) rubric provides guidance on course introduction, learning objectives, assessment/measurement, instructional materials, learning activities/interaction, course technology, learner support, and accessibility/usability. Perhaps most relevant for the present study, the standards for course technology include specific recommendations on recruiting multiple technologies and using digital tools that support course objectives and promote engagement as well as learning.

Instructors often turn to such frameworks as QM when designing their online courses, to bolster student engagement and success. In a study on instructors’ facilitation strategies, Martin et al. (2018) found that students most appreciated timely feedback and responses while strategies such as interactive syllabi and live sessions were rated lower. Ralston-Berg et al. (2015) analyzed students’ perceptions of online course quality to see if they aligned to QM standards*.* Student perceptions mostly aligned, though some aspects were rated as less important by students. Students viewed clear instructions, logical navigation, and readily available technology as most important while learning activities that encouraged interaction were not as important. Such research is critical as students’ satisfaction is connected to other educational outcomes, such as to their retention and motivation (Lei & Yin 2020).

Though instructors’ facilitation strategies in online courses are critical for meeting quality standards, the conditions of the COVID-19 pandemic presented a unique challenge. While some instructors may have been aware of frameworks such as *Quality Matters,* others may have had little to no training in online pedagogy. With limited time to make the move, and sometimes limited institutional support (some colleges may have never offered online courses before), it can be difficult to adhere to best practices.

### Online courses and psychological variables

It is possible then that students enrolled in online courses that arose unexpectedly and may not have been designed with as much focus on frameworks such as QM. Furthermore, research during the COVID-19 pandemic has suggested that students are experiencing new situations and stressors that contribute to sleep issues, trouble focusing, fewer social interactions, enhanced concern with academics, and more (Son et al., 2020). Accordingly, the experiences of students in these online courses may vary from typical circumstances and be intertwined with individual differences in unique ways. To highlight these potential relationships, we explored several individual characteristics of student learners: engagement, motivation, procrastination, stress, and self-efficacy.

Research has found that engagement is vital for achieving positive outcomes in online courses (e.g., Czerkawski & Lyman III, 2016). New circumstances and stressors during the pandemic may lead students’ engagement to fluctuate. There are design options that instructors can use to facilitate course engagement (e.g., required meetings with that instructor) but also behaviors that students can perform to enhance their own engagement (e.g., keeping up on their readings or participating in discussions).

Students’ motivation may also waver during these unusual times. Extrinsic motivation (e.g., get a passing grade) often drives practices such as studying, and may be exacerbated during a pandemic as students face new stressors. Research has shown connections between intrinsic motivation and online course participation (Xie et al., 2006), and researchers agree that personal initiative to do well drives success (Mandernach et al., 2006).

Procrastination may also fluctuate under these circumstances. On one hand, students may be at home with fewer activities and social engagements, and thus may complete coursework sooner. On the other hand, students may be facing challenges such as sharing computers and caring for sick family members, which could enhance procrastination. In general, procrastination is a common practice amongst college students, and lower levels contribute to student success (You, 2015).

Additionally, stress has taken on new forms, small and large: financial concerns, cancelled social engagements, health issues, and more. When surveyed, college students reported increased stress and anxiety during the pandemic, with less than half believing they could adequately cope (Wang et al., 2020). Some research has suggested that stress rose in college students during spring semester 2020 but may have returned to pre-pandemic levels during fall semester 2020 (Charles et al., 2021). That said, stress varies by circumstance. For fall 2020, schools adopted varying modalities, though the virtual approach was still most common (Walke et al., 2020). Housing and campus situations were similarly variable, as was the timeline for receiving updates for the semester. In addition to the broader context, daily hassles such as changing deadlines and cancelled events also influence perceived stress for students.

Lastly, self-efficacy, or a person’s belief in their own capacity to successfully execute a behavior, may vary as students encounter different resources than they may be used to, such as no library or computer lab next door. This may influence the confidence they have in themselves to do well. Furthermore, online courses may require more self-efficacy than in-person courses, given the self-guidance required to advance through coursework. Like the other variables, research has shown a connection between high self-efficacy and academic success (e.g., Bradley et al., 2017).

The preceding variables all represent individual differences between students. These factors can be interrelated, such as positive relationships between motivation and self-efficacy (Salazar & Hayward, 2018) or engagement (Martin & Bolliger, 2018) and a negative relationship between self-efficacy and procrastination (Wolters et al., 2017). In addition to this set of variables, we wondered how cognitive load might vary during these times. Though cognitive load often relates to grades (e.g., Sage et al., 2020), cognitive space for both students and teachers may be occupied with pandemic-related stressors. This could influence students’ perceptions of effort and course difficulty as well as actual course difficulty levels.

A final variable we considered in the context of this pandemic, as more of an outcome variable, was the level of isolation that was felt. At various points in recent months, students have experienced stay-at-home orders, quarantined, and self-isolated. In short, they are experiencing a starkly different social landscape than they are probably used to in college where social exchanges abound. Feelings of isolation can influence other outcomes such as dropout rates and persistence in a course (e.g., Yuan & Kim, 2014). Research during the COVID-19 pandemic has been contradictory, suggesting simultaneously that loneliness has not increased (Luchetti et al., 2020) and has increased (Lee et al., 2020) at different times. Online classes could exacerbate these same isolating experiences or potentially provide a social outlet for students.

### Smartphones for learning

With the move online, there was a quick need for students to use their personal devices to engage in coursework. As previously mentioned, smartphone ownership is near universal among the college student population (e.g., Sage et al., 2020). Laptop ownership is similarly high at above 90% (Gierdowski, 2019; Sage et al., 2020). In an NPR article written during the pandemic (Nadworny, 2020), it was speculated that computer availability declined for college students. Even if students had their own laptops, the conditions necessitated by the pandemic might have led students to share devices with others in their household working remotely or attending virtual school (Chiner et al., 2021). Furthermore, even if the house had multiple computers, streaming video or accessing large files across multiple computers in the home can be challenging and create bandwidth issues, particularly if all devices are on the same WiFi network. Chiner et al. (2021) reported that 31.5% of students (at a Spanish university during the pandemic) always or often had a poor Internet connection during online learning experiences. This could lead to students being dropped from live class sessions or having trouble up/downloading files. Using smartphones to engage in coursework, particularly if equipped with unlimited data, may be one method to circumvent the need to share a WiFi network that is overburdened. Rahiem (2021) investigated device sharing within a sample of university students in Indonesia during the COVID-19 pandemic, reporting that a majority of students used their phone’s Internet access and data plan to access materials and to tether their laptop to the Internet. Students may not have encountered the same pressure to share smartphones with other household members as well. Rahiem (2021) also reported that, while about 30% of students were sharing laptops with other family members, all students had smartphones.

Mobile devices such as smartphones do have promise for education given their unique features, such as personalized interfaces and social applications. Growing literature has endorsed smartphone use in education. Anshari et al. (2017) surveyed college students, finding that students often used smartphones to retrieve digital course materials and browse the Internet for relevant information. Interestingly, students noted using the Internet more often on their smartphones than laptops. Students mentioned such reasons as convenience and portability, while also noting the benefit of smartphones for instant communication with teachers and classroom peers. Agreeably, Sage et al. (2020) reported that reviewing flashcards on a smartphone was just as effective as reviewing flashcards on a laptop or paper cards. Sage and colleagues (under review) further reported that smartphones were just as effective as laptops for completing basic academic tasks like sending an email to a professor. In that research, students reported believing that smartphones were more effective than laptops for completing polls or surveys in class, receiving assignment reminders, and reviewing communication from peers and professors. They complimented smartphones for their size, convenience, and easy access to resources. Agreeably, Crompton and Burke (2018) have emphasized that humans are increasingly comfortable with small screens given their regular use in everyday life.

On the other hand, many learners and instructors believe that smartphones do not facilitate learning. Smaller screens may challenge learners in new ways. Kim and Kim (2010) noted the possibility of enhanced cognitive load from such traits as small font size. Kim et al. (2019) reported that phones distract students from their classwork every 3–4 min. Tossel et al. (2015) studied smartphone use in college students without prior smartphone experience. Though students had viewed phones positively relative to education prior to the study, they viewed phones as more of a distraction by the end. Like other research, students did label the devices as useful for retrieving course materials and communicating with others. And though Sage et al. (under review) found the aforementioned positive characteristics of smartphones, they also reported that students saw more educational value in laptops over smartphones and believed laptops were superior for interactive activities, research, accessing some course materials, and note-taking. They complimented laptops for their ability to login to class meetings and complete assignments.

When focused specifically on online coursework, the advantages of the bigger screen afforded by the computer may be intuitively clear: more space to view your instructor and class during live sessions, a larger keyboard for typing, and easier navigation with multiple browser windows open. However, smartphones may also have unique functionality to assist in online coursework, as they can provide a constant connection to peers, facilitate groupwork and communication given many apps and notifications, and have an internet connection that relies less on a home WiFi network. Al-Hariri and Al-Hattami (2017) investigated the relationship between students’ use of both phones and laptops with academic achievement. They discovered a positive relationship between these variables, citing such reasons as the role of technology in encouraging independent, self-directed learning and in expediting student collaboration. Thus, both devices may play into students’ experiences with their online courses.

### The current study

This study focuses on college students’ technology choices, experiences, and perceptions in an online course during the pandemic. Our work expands current literature in several ways. First, we contribute to the growing literature on the educational experience during the COVID-19 pandemic. Second, we add to the literature on online classes by focusing on the physical technology used. Smartphones are an understudied device in students’ learning (see meta-analyses: Delgado et al., 2018; Sung et al., 2016). Third, we investigate student outcomes as well as various individual difference measures to see where relationships might lie. In addition, we employ both quantitative and qualitative data approaches to provide a richer data set when drawing conclusions.

### Research questions and hypotheses

#### How do student outcomes, individual differences, device, and cognitive load relate?

As our primary analysis, we sought to reveal key relationships between our variables. Student outcomes were operationalized as current grade, satisfaction, and isolation felt in the course. Individual differences included engagement, motivation, procrastination, stress, and self-efficacy. Device was operationalized as the proportion of coursework completed on laptops versus smartphones. Cognitive load included students’ perceptions of course difficulty and effort expelled.

In this analysis, we expected that more smartphone use might be related to poorer student outcomes, more negative scores on the individual difference measures, and higher cognitive load. This seemed possible given certain affordances of the smartphone, such as more difficulty focusing on and reading a smaller screen. The one exception was for isolation felt, where it seemed possible for smartphone use to be a boon since it is a social device. We additionally expected our individual difference measures to correlate to one another as well as meaningfully relate to student outcomes and cognitive load in ways generally consistent with the literature described earlier. Though mean levels of these items might all vary during a pandemic, we believed that the underlying relationships would hold true.

#### What variables predict student outcomes in these online courses?

As a secondary analysis to look at these data from a different angle, we sought to ascertain whether device and the individual difference measures meaningfully predicted the three outcome variables. We believed that smartphone use might be a negative predictor of course grade and satisfaction while laptop use might be a positive predictor of student outcomes, given the challenges created by using smaller devices for online coursework. It also seemed possible that students’ choice in technology may influence their feelings of isolation. Smartphones may enhance feelings of social connection given the constant notifications from various social apps, and thereby their use might predict lower feelings of isolation. We also believed that the individual difference measures would contribute to the variance in these outcomes, consistent with past research (e.g., Bradley et al., 2017; Czerkawski & Lyman III, 2016).

#### What are students’ experiences with, and perceptions of, these technologies for online courses?

This section was exploratory, to provide qualitative supporting data on students’ experiences with these technologies and their associated perceptions. Thus, we did not form specific hypotheses. Questions focused on students’ experiences with laptops and smartphones for their online courses, as well as what they liked and disliked about their technology and online courses more generally.

## Method

### Participants

Ninety-six undergraduate students (57.3% female, 40.6% male, 2.1% other; *median age* = 19 years) at a Northwestern liberal arts college in the United States completed this study for course credit. All students were enrolled in a *General Psychology* course in fall semester 2020. Students were drawn from five sections of this course that had shifted from in-person to online prior to the start of the semester, given the ongoing pandemic and health precautions. Students reported their race and ethnicity as 71.9% White, 12.5% Hispanic or Latinx, 12.5% Black, 11.5% Asian, 8.3% mixed race, 5.2% Native Hawaiian or Other Pacific Islander, 3.1% American Indian or Alaskan Native, and 1% other. Students were primarily first-years and sophomores (92.7%). Two other individuals participated, but their data were excluded due to misunderstanding or misreading part of the survey.

As additional context, the college in question had shifted to an online class model for approximately half of spring semester 2020, after local COVID-19 cases appeared and a stay-at-home order was issued. The college initially planned for in-person classes to resume for fall semester 2020. Fluctuations in local COVID-19 case numbers and many considerations throughout summer led to shifting back to primarily virtual courses for fall semester 2020. Given that the official announcement preceded the semester start date by just a few weeks, many faculty and students adjusted and shifted plans quickly. Most students lived off-campus during the semester, but a limited number were provided with on-campus housing.

### Measures

#### Technology and course survey

Students answered questions about their experience in their *General Psychology* course and the technology used. They indicated what percentage of their work for class (attending class, reviewing slides, etc.) occurred on laptop and desktop computers, smartphones, tablets, or other devices. They further indicated how satisfied they were with their online course, as well as how difficult and effortful they felt it to be, on 0–6 scales. Similar satisfaction questions, and 7-point scales, are typical of learning research (e.g., Kablan & Erden, 2008; Moreno & Valdez, 2005; Vandewaetere & Clarebout, 2013). These self-report questions on cognitive load (effort, difficulty) have been found valid and reliable (e.g., Paas et al., 2003; Teo et al., 2003).

To explore satisfaction more deeply, students described what they liked, disliked, and would improve about their experience with technology in their course. Furthermore, they listed drawbacks and perks of using laptop computers and smartphones for classes. We selected these two technologies to explore further given past research showing near universal ownership of these technologies compared with less-than-half ownership of other technologies, including both tablets and desktop computers (e.g., Sage et al., 2020). Students also listed one thing that is good about doing activities online versus in-person and vice versa.

Lastly, students noted the approximate number of hours per week spent on their *General Psychology* course in addition to their current course grade. Students then moved a bar to indicate how isolated they felt in their online coursework on a 0 (not at all)–100 (very) scale. Single items have been previously used to measure feelings of isolation in adults (e.g., Sansoni et al., 2010).

#### Engagement

To indicate their course engagement, students described the extent to which particular behaviors, thoughts, and feelings described them in relation to their *General Psychology* course (questionnaire adapted from Dixson, 2010). They responded to 19 statements, such as “Finding ways to make the course material relevant to my life” and “being organized,” on a 1 (not at all characteristic of me) to 5 (very characteristic of me) scale.

#### Academic motivation

The Motivated Strategies for Learning questionnaire (Pintrich & DeGroot, 1990; Pintrich et al., 1993) measured students’ academic motivation in their course. This questionnaire contained 44 statements on a 1 (not at all true of me) to 7 (very true of me) scale. Example questions include “I prefer class work that is challenging so I can learn new things” and “I like what I am learning in this class.”

#### Procrastination

The procrastination scale (Lay, 1986) included 20 questions on a 1 (extremely uncharacteristic) to 5 (extremely characteristic) scale to measure students’ tendency to postpone more important for less important activities. Example questions include “A letter may sit for days after I write it before mailing it” and “I usually buy even an essential item at the last minute.”

#### Perceived stress

The perceived stress scale (Cohen et al., 1983) included 10 questions on a 1 (never) to 5 (very often) scale and indicated the degree to which students felt their lives were stressful. Example questions include “In the last month, how often have you been…. …upset because of something that happened unexpectedly?” and “…able to control irritations in your life?”.

#### Self-efficacy

The self-efficacy scale (Scholz et al., 2002; Schwarzer & Jerusalem, 1995) included 10 questions on a 1 (never) to 4 (exactly true) scale, and measured students’ perceived ability to handle daily hassles. Example questions include “If I am in trouble, I can usually think of a solution” and “I can usually handle whatever comes my way.”

#### Demographics

Students indicated gender, class year, age, race and ethnicity.

### Procedure

Students reviewed a consent form and checked a box to indicate consent. Students completed the survey in the preceding order. The debriefing page then thanked students and provided information on how to access mental health support at the college.

### Data analysis approach

Correlational analyses addressed hypothesis 1 about relationships between variables. A stepwise regression analysis addressed hypothesis 2, to indicate if the individual difference measures and device predicted student outcomes in any meaningful way. Lastly, to answer the third research question, we performed exploratory quantitative and qualitative analyses on the survey data. To code open-ended questions, two researchers reviewed student responses and created a list of themes. Themes such as enjoying a technology for its portability and disapproving of it for its distractions emerged. Two researchers met to discuss these themes and potential interpretations (Bogdan & Bilden, 2007). As data analysis continued, these themes were expanded, developed, and merged as necessary (Glaser & Strauss, 1967). We then coded responses to their respective themes. Inter-rater reliability for all coding was above 90%.

## Results

### How do student outcomes, individual differences, device, and cognitive load relate?

Averages and standard deviations for the variables appear in Table 1. Overall, students were earning high course grades (a B average) and feeling somewhat satisfied, but also feeling somewhat isolated, in their course. The individual difference measures showed that the current sample had middle to high engagement, motivation, and self-efficacy, and middle to low procrastination and stress levels. Laptop use was far more common than smartphone use for coursework. The unaccounted percentage was primarily filled by students using tablets instead of laptops or smartphones. Additionally, students felt that course difficulty was around the midpoint, and that some effort was required to complete their courses.

**Table 1:** Means and standard deviations

	Mean (SD)	Scale range
Grade (% grade)	85.8% (11.8%)	0–100%
Satisfaction	4.11 (1.36)	0–6
Isolation felt	65.05 (28.96)	0–100
Engagement	3.41 (.59)	1–5
Motivation	4.90 (.71)	1–7
Procrastination	2.88 (.63)	1–5
Stress	2.25 (.72)	1–5
Self-efficacy	3.06 (.41)	1–4
Laptop use (% of time)	88.6% (20%)	0–100%
Smartphone use (% of time)	5.1% (9.2%)	0–100%
Difficulty	2.75 (1.22)	0–6
Effort	3.98 (1.14)	0–6

As our primary analysis, we conducted correlations to reveal relationships amongst variables. As per Table 2, hypothesis 1 had some support in that numerous variables were interrelated. However, the proportion of coursework on a particular device did not relate to the other variables. The one exception was a negative correlation between smartphone use and isolation felt; the more someone proportionally used their smartphone, the less isolation they felt. Other correlations were generally as expected. When looking at the outcomes of grade, satisfaction, and isolation: A higher grade related to higher satisfaction, engagement, and motivation as well as lower feelings of isolation, procrastination, and stress. Course satisfaction was similarly connected to these variables, with the addition of being negatively correlated to perceived course difficulty. Isolation was connected to smartphone use, and had a strong positive correlation with perceived stress.

**Table 2:** Correlational analyses

	1	2	3	4	5	6	7	8	9	10	11	12
1 Grade		.43***	− .23*	.50***	.23*	− .35***	− .31**	.06	− .02	.05	− .19	− .12
2 Satisfaction			− .12	.47***	.43***	− .31**	− .30**	.12	.02	− .12	− .35***	− .05
3 Isolation				− .19	− .10	.16	.43***	− .15	− .05	− .25*	.10	.11
4 Engagement					.62***	− .50***	− .31**	.32**	.01	.07	− .16	− .07
5 Motivation						− .39***	− .07	.24*	.10	− .19	− .14	.04
6 Procrastination							.37***	− .31**	.08	.06	− .05	− .08
7 Stress								− .50***	− .01	− .04	.08	.11
8 Self-efficacy									.04	.05	.07	< .01
9 Laptop use										− .32**	− .01	− .09
10 Smartphone use											− .01	.02
11 Difficulty												.52***
12 Effort												

*p ≤ .05, **p ≤ .01, ***p ≤ .001

### What variables predict student outcomes in these online courses?

Stepwise regression was conducted on the three variables defined as outcomes: current course grade, satisfaction, and isolation felt in the course. The five individual difference measures (step 1) and two device variables (step 2) were entered as predictors. Adjusted R^2^ values are reported along with standardized coefficients with their significance. It can also be noted that, though these variables were somewhat correlated with each other as evidenced in Table 2, multicollinearity was not indicated as a concern; the VIF was close to 1 in all cases (a value of 5–10 is a general standard for multicollinearity issues in the literature, e.g., Akinwande et al., 2015; Yoo et al., 2014). For course grade, the first model explained 29.8% of the variance, *F*(5,85) = 8.64, *p* < 0.001, with engagement, β = 0.50, *t* = 4.05, *p* < 0.001, stress, β = − 0.24, *t* = − 2.21, *p* = 0.03, and self-efficacy, β = − 0.25, *t* = − 2.32, *p* = 0.02, emerging as predictors. Step 2, or device used, did not explain any additional variance. For course satisfaction, the first model explained 25% of the variance, *F*(5,81) = 6.74, *p* < 0.001, with motivation, β = 0.28, *t* = 2.31, *p* = 0.02, and marginally stress, β = − 0.23, *t* = − 1.93, *p* = 0.057, and engagement, β = 0.25, *t* = 1.91, *p* = 0.059, as predictors. Step 2 again did not explain additional variance. For isolation felt, the first model explained 16.1% of the variance, *F*(5,88) = 4.58, *p* = 0.001, with only stress emerging as a significant predictor, β = 0.49, *t* = 4.18, *p* < 0.001. In step 2, smartphone use also emerged as a significant predictor, β = − 0.32, *t* = − 3.13, *p* = 0.002, resulting in a significant F change. The model now explained 23.1% of the variance in isolation felt, *F*(2,86) = 5.00, *p* = 0.009.

### What are students’ experiences with, and perceptions of, these technologies for online courses?

As previously mentioned, students overwhelmingly used laptops over other technologies for their coursework. Almost all students used laptops heavily, with the most common laptop models being MacBooks or HP models, with 12–15’’ screens. Smartphones were used by almost 60% of students for coursework and were most commonly iPhones. Tablets, typically iPads, were used by about 12.5% of students. Desktop computers were rarely used, with only four students in the sample reporting any time on a desktop computer. Students labeled many advantages and drawbacks to both laptops and smartphones (see Tables 3, 4). They shared the advantages of ease of use and portability, but laptops additionally were multi-functional with large screens and more storage. Laptops had issues with WiFi and battery to a higher degree, while students found notifications/distractions as more troublesome on their smartphones along with the smaller screen and general difficulty with completing academic tasks.

**Table 3:** Advantages of laptops and smartphones (N = 96)

	Laptops % of sample	Smartphones % of sample
Ease of use	71.9	53.1
Portable	45.8	60.4
Multi-functional	43.8	–
Large screen	20.8	–
More storage	16.7	–
No perks/none listed	3.1	13.5

Responses often contained multiple advantages, and were thus categorized in multiple categories

**Table 4:** Drawbacks of laptops and smartphones (N = 96)

	Laptops % of sample	Smartphones % of sample
Faulty WiFi connection	20.8	6.3
Battery/charger issues	16.7	3.1
Notifications/distractions	15.6	39.6
Slow/can get overwhelmed	14.6	–
Not portable	14.6	–
Lack of human connection	8.3	–
Small screen	–	29.2
Academic tasks are difficult	–	28.1
Hard multi-tasking	–	13.5
Less storage	–	8.3
Typing difficulties	–	8.3
No drawbacks/none listed	13.5	5.2

Responses often contained multiple drawbacks, and were thus categorized in multiple categories

Students were generally satisfied with their online course, as indicated earlier by averaging 4.11 (*SD* = 1.36) on a 0–6 scale. The reasons behind their satisfaction ratings are provided in Table 5. Generally, students appreciated their technology being easy to use and streamlining their academic tasks but struggled at times with issues of engagement and device performance.

**Table 5:** Reasons for student satisfaction with technological device (N = 96)

	Satisfied by these features % of sample	Dissatisfied by these features % of sample
Streamlining Academic Tasks	43.8	5.2
Ease of Use	34.4	9.4
Student Engagement	19.8	39.6
Portability	16.7	1.0
Promotion of Deep Learning	11.5	7.3
Device Performance	7.3	37.5
Cost	1.0	1.0
Health Implications	–	8.3

Responses often contatined multiple reasons, and were thus categorized in multiple categories

Students were asked about ways to improve their online learning experience. Though a large portion of the sample said that no improvements were needed (29.2%), other students gave suggestions for fixing technology problems to improve their quality and performance (34.4%, such as using a different platform for live sessions), improving the general learning environment and participation (15.6%, such as by eliminating distractions), and making course information more available and clearer (10.4%, such as with organization and reminders). Some students (10.4%) also indicated a desire to have more student and professor training on technology and applications used.

Lastly, students noted course characteristics that were better online versus in-person and vice versa. Students reported preferring in-person classes for the social interaction (39.6%), engagement (27.1%), ability to focus/less distractions (20.8%) and easier communication (10.4%). But online classes often were preferred for their convenience (22.9%), self-paced nature (16.7%), comfortable environment (14.6%), and additional tools for learning (12.5%).

Overall, students seemed to have a good sense of why they chose a particular technology for their coursework, were generally content with that choice (though they saw room for improvement overall for online classes), and were aware of the potential drawbacks.

## Discussion

We investigated how student outcomes, individual differences, devices, and cognitive load relate in the context of students taking online courses during the COVID-19 pandemic. Though choice of device did not predict course grade or satisfaction nor correlate to most variables, the proportion of time students spent using smartphones for their classes was negatively correlated to isolation felt. Though phones were only used for a small proportion of their classwork, students who did use them more frequently reported feeling less isolated in their course. Agreeably, when a regression was run, the proportion of time students spent using their smartphones individually explained an additional ~ 7% of the variance in isolation felt. When considering the relation between smartphone use and social connection, it might be noted that we only measured smartphone use in the context of online coursework. This strategy was opposed to measuring broader smartphone use in daily life. This begs the question then of how these typically brief encounters with smartphones during coursework may have reduced feelings of isolation. Informal conversations with the instructors of *General Psychology* suggested that smartphones were sometimes called upon for class interactions such as online review games. Though students can often use their laptop for those types of activities as well, using one’s smartphone might offer something new and engaging for the learner. It might also lead students to brief social encounters during class (e.g., returning text messages or seeing social media notifications).

Despite the ubiquity of laptops, the proportion of time students spent on their laptops for coursework did not relate to other variables nor predict any outcomes. These findings have implications for the technology recommendations that educators make to students. Perhaps both laptops and smartphones play key roles in students’ academics in this context, with laptops being preferred by many students for coursework but the smartphone also providing feelings of social connection.

Notably, this result may align well with one component of the *Quality Matters* framework mentioned earlier. That framework includes a technology item on its rubric, which gives recommendations for using multiple digital tools and ensuring that those tools align with course objectives and support engagement and learning. Agreeably, Ralston-Berg et al. (2015) found that students appreciated readily available technology. Perhaps using both laptops *and* smartphones for coursework can help in meeting these standards and create the best overall experience for students. The smartphone can be used when the laptop fails in some way (whether that be poor WiFi connection, battery issue, or something else) and it can be used to socially connect with others even while actively doing other coursework on one’s laptop. Students in our study reported the biggest pros of both smartphones and laptops as their portability and ease of use. Perhaps tellingly, for drawbacks, some students reported being distracted by notifications on the smartphone but lacking human connection on their laptops. The laptop and smartphone together might create a balanced set of affordances for the learner, each meeting slightly different needs.

Furthermore, we noted numerous correlations between outcomes and individual differences, in mostly predictable ways. For instance, grades were higher when engagement was also higher and procrastination was lower. These correlations match the literature described earlier and show that the same relationships apply during the context of a pandemic. Mean levels of the individual difference variables also suggested that students were not behaving or functioning in unusually poor ways; for instance, stress was below the neutral mid-point and self-efficacy was rather high. Perhaps as Charles et al. (2021) suggested, variables such as stress may have returned to their pre-pandemic level for fall semester. Interestingly, cognitive load only correlated to students’ satisfaction. Other research has shown links between variables and cognitive load, such as to a grade on a quiz (e.g., Sage et al., 2020). In the present study, it seems possible that the *General Psychology* course was simply not very difficult and thus students were not typically over-burdened by the course. In support of this, students averaged a B grade and rated difficulty around the neutral midpoint. Further, when asked how many hours they spent on their *General Psychology* course, students reported an average of only 4.82 h/week (*SD* = 2.42). This number was about half of the expected amount; the general standard at the college was 3 h of class time on top of a minimum of 6 h out-of-class time per week. It seems plausible that the course was designed to be easier than typical during this semester, to account for the course unfolding during a global pandemic and students and teachers alike experiencing new challenges.

Interesting correlations did emerge around isolation felt. Higher feelings of isolation related to lower grades and more stress. And though mean levels of the individual difference measures leaned in positive directions, the mean level of isolation was rather high—about 65 on a 100-point scale. Students often report feeling isolated or disengaged in online courses (Patel, 2021). However, building community can help counteract these feelings (Phirangee & Malec, 2017). Though students might not always see the utility of interactive learning activities (Ralston-Berg et al., 2015), they may play an important function for building students’ sense of connection, and perhaps especially amidst a context that consists of other isolating practices such as quarantining. Educators must be deliberate in their approaches to building community in the online setting. Online learners seek comfort; they want a comfortable class atmosphere alongside opportunities for learner-to-learner interactions (Van Wart et al. 2020). Such online social comfort acts to decrease students’ feelings of anxiety. Recent research during the pandemic has suggested that students report feeling more connected to the community when video conferencing is used, given the face-to-face interaction (Boardman et al., 2021). Intentional scaffolding techniques are also beneficial (Patel, 2021). Overall, instructors must consider ways to increase feelings of community in the online classroom.

When predicting student outcomes, stress emerged as a consistent predictor across all three regression analyses. This finding is perhaps unsurprising given the current global context and past research, though recall that stress was not particularly high in our sample. Even if on the lower side, stress can still negatively influence outcomes such as student retention (Saunders-Scott et al., 2018), as it may draw attention away from coursework and have ramifications for mental health. On top of that, the pandemic may be altering the stressors affecting students. As suggested by past research (Hintz et al., 2015), stress may be an important target for student intervention in the current context. Agreeably, Copeland et al. (2021) indicated that students enrolled in campus wellness programs experienced fewer internalizing and attention problems related to the pandemic relative to those not enrolled in wellness programs.

Expectedly, student engagement also predicted course grade and satisfaction. Though engagement might look different in online courses relative to in-person courses (e.g., logging into virtual lectures and participating in discussion boards), past research has shown that it is consistently linked to student success (Czerkawski & Lyman III, 2016). In the current context, students may need to push themselves more to engage, with classes on the screen, distracting learning environments at home, and other new stressors. But if successful, it positively impacts their course experience. To encourage this engagement, teachers might consider the *Quality Matters* framework mentioned earlier. Though the quick shift to the screen may have left limited time for preparation, picking out strategies (e.g., Martin et al., 2018) aimed at boosting student engagement may encourage student success and retention.

### Practical implications

Educators might consider methods for using different technologies in their course activities. This may be increasingly important in online courses, to help vary the format and engage students. For example, if smartphones help lower feelings of isolation and assist communication, then educators might encourage students to create chat spaces and collaborative documents on their phones for groupwork, engage in live review games with each other, or complete online surveys (e.g., Remón et al., 2017). Also, this recommendation does not apply only to online education. Given that almost all students have a smartphone, educators might consider how to incorporate them into their in-person classes as well. Though professors often have a distaste for smartphones in their classrooms given the potential for distraction, there are ways to use them effectively for learning (Bayless et al., 2013).

Colleges might also consider investing in additional technology training and online course workshops for their faculty and students. Especially when course modality shifts so suddenly, additional workshops by the IT department can facilitate a successful transition and bring both students and faculty up-to-speed. Such workshops could cover topics ranging from specific programs used by the college to overarching frameworks such as *Quality Matters*. Some students explicitly commented on this desire, and many others expressed how improvement to technology would boost their satisfaction with their online course.

In addition, colleges should consider that some students may not have regular access to laptops or smartphones. This may be exacerbated by such challenges as sharing devices at home. Though some campuses have rental programs, others do not, or students are currently not near their campus. And some students simply cannot afford to purchase such devices (Hargittai, 2010). Thus, colleges might consider ways to assist students needing reliable technology, such as need-based grant monies or mailing temporary rental devices. Readily available technology was deemed a critical feature of online courses by students (Ralston-Berg et al., 2015), and such options might reduce students’ stress as well.

Lastly, colleges might also consider how to better target stress management with students as well as how to reduce their feelings of isolation. During unprecedented times, such as the current pandemic, colleges need to be extra creative in their approach. Though some campuses shuttered their buildings, counseling centers can still offer virtual services and resources for students. Helpful suggestions might include having professors individually identify students in their classes that are struggling, providing self-care information on syllabi, and scheduling virtual mental health sessions. Weekly stress reduction activities could be offered (e.g., virtual yoga or meditation) as well as opportunities to socially engage with others (e.g., virtual tutors or just conversations on current events). Helpful workshops could even be encouraged with course credit. Lederer et al. (2021) warned of not letting such services diminish with the budget cuts inflicted during financial hardships (such as those caused by the pandemic), and similarly encouraged technology-based services and a supporting role for faculty.

### Limitations and future directions

This research had several key limitations. First, the context is niche. We analyzed these variables during a global pandemic, which is a rare event. That said, the findings can be considered as a jumping off point for future research. For instance, we found that stress was a consistent predictor of student outcomes. Even when schools have returned to their in-person state, offering virtual counseling services may continue to benefit students. Future research could investigate the utility of such services in addition to typical on-campus offerings. Additionally, smartphones might be able to enrich online classes in new ways. Future research could include smartphone assignments in class, to see if that modality is beneficial for students’ learning and/or well-being. Though many students used their phones for coursework to some degree in the present study, they were used minimally. It would be interesting to manipulate the amount of time on the smartphone for coursework to see if there was a helpful versus hindering threshold as well as determine the specific activities that are better suited for the laptop versus smartphone. These variables could also be examined in unison—how might device choice influence the stress a student feels towards their coursework? Perhaps a blend of technology could reduce stress for the student; one technology could be a ‘back-up’ to the other one and/or act to meet different student needs.

Furthermore, we assessed student outcomes via several global measures—current course grade, a satisfaction rating, and an isolation rating. Future research could recruit other measures to acquire a more robust understanding of the relationships described here, such as looking at overall semester GPA or specific types of assignments (e.g., exams versus papers). Isolation could also be examined more thoroughly, both quantitatively and qualitatively, such as by probing reasons for students’ isolation ratings. Lastly, additional individual difference measures could be investigated. We selected variables that seemed like they could fluctuate during the present time, but many others could show variability as well.

## Conclusion

Many colleges quickly moved their coursework online during the COVID-19 pandemic. Students and instructors alike faced new challenges and academic experiences. Our results suggest that students may benefit from using multiple technologies in their online coursework. Though the laptop was the tried-and-true resource for completing coursework, students seemed to reap some benefit from using their smartphones too; it predicted lower feelings of isolation. Phones have a reputation for being social devices and may help students feel more connected. Stress also emerged as a consistent predictor of student outcomes. Though their reported stress level was not unusually high, the type of stress experienced during the pandemic may have been new and unexpected. Future research might further examine the interplay between device and stress as well as continue to explore the smartphone dynamic in the virtual classroom.

## Data Availability

Data can be made available by individual request to the corresponding author.

## Abbreviations

COVID-19: Coronavirus disease 2019
QM: Quality Matters
